# Therapeutic effects of exercise-accompanied escitalopram on synaptic potency and long-term plasticity in the hippocampal CA1 area in rats under chronic restraint stress 

**DOI:** 10.22038/IJBMS.2022.66718.14629

**Published:** 2022-12

**Authors:** Mahshid Zamani, Maryam Radahmadi, Parham Reisi

**Affiliations:** 1 Department of Physiology, School of Medicine, Isfahan University of Medical Sciences, Isfahan, Iran

**Keywords:** Escitalopram, Exercise, Long-term potentiation, Neuronal plasticity, Stress

## Abstract

**Objective(s):**

Administration of antidepressants and exercise are among the therapeutic approaches to chronic stress. Therefore, this study compared the therapeutic effects of different doses of escitalopram, exercise, and exercise-accompanied escitalopram on synaptic potency and long-term plasticity in the hippocampal CA1 area in rats under chronic restraint stress.

**Materials and Methods:**

The rats were allocated to different groups. The chronic restraint stress (6 hr/day) continued for 14 days. Injection of escitalopram (10 and 20 mg/kg) and treadmill running (1 hr/day) were performed after the stress induction. The input/output (I/O) functions and LTP induction were evaluated in the hippocampal CA1 area.

**Results:**

The fEPSP slope and amplitude after the LTP induction significantly decreased in the chronically stressed group. However, the serum corticosterone levels had significant enhancement in this group. In addition to serum corticosterone levels, the fEPSP slope and amplitude after the LTP induction were enhanced by exercise, escitalopram 20 mg/kg alone, and exercise-accompanied escitalopram 10 and/or 20 mg/kg in chronically stressed groups.

**Conclusion:**

Overall, chronic stress impaired synaptic potency and long-term plasticity. These impairments were effectively reversed by exercise, escitalopram 20 mg/kg alone, and exercise-accompanied escitalopram 10 and 20 mg/kg. However, escitalopram 10 mg/kg alone could not alleviate the memory deficits in chronically stressed subjects. Therefore, exercise with both doses of escitalopram seems to have had additive effects on chronic stress conditions.

## Introduction

Chronic stress leads to exacerbation and acceleration of some mental illnesses, such as depression and anxiety via the hyperactivity of the hypothalamic-pituitary-adrenal (HPA) axis (1). Also, the elevated levels of serum cortisol under chronic stress exert neurotoxic effects on the hippocampal neurons, which result in reduced functions of various mechanisms, such as neurogenesis, synaptogenesis, dendritic spines, long-term potentiation (LTP), and brain functions, as well as increased neural apoptosis (1-3). Numerous pieces of evidence have shown that hippocampal LTP is the electrophysiological basis for learning and memory (4). Therefore, it has been used to assess experience-dependent plasticity or memory (5).

Nowadays, the treatment of stress has become very important in societies as it is involved in various mood and brain disorders including, anxiety, depression, and cognitive impairments (1). Antidepressants are used for treatment of stress and potential brain dysfunctions that are caused by it (6). Selective serotonin reuptake inhibitors (SSRIs) are a class of first-line antidepressants that are recommended for improving the state of stress-associated disorders (e.g., depression and anxiety) (6, 7). As an important mechanism, they enhance the net serotonergic transmission by blocking the presynaptic serotonin uptake sites (7). Escitalopram (S-enantiomer of citalopram), the most common SSRI in the treatment of stress, anxiety, and depression (8) is more potent than citalopram (9). In different studies, escitalopram has either ameliorated or decreased memory, and in some cases, it had no impact on memory (10). Therefore, the memory-associated effects of escitalopram remain controversial. Drug therapy has different side effects and a slow response time; therefore it might need an extra treatment regimen to be effective (11). Therefore, further safe treatment procedures are required to reverse the effects of memory disorders that are induced by chronic stress. 

According to previous studies, aerobic exercise is a non-pharmacological strategy for improving various brain functions, such as cognition and mood (12, 13). Similar to antidepressants and anxiolytics, exercise has also beneficial effects (14) and suppresses the decreased proliferation of hippocampal cells due to chronic stress or corticosterone administration (15). Also, regular exercise enhances angiogenesis, neurogenesis, and synaptic plasticity in the brain (16, 17). Different protocols like pharmacological manipulation and exercise could modulate LTP (18). Hence, in the treatment of stress and stress-related disorders, such as memory impairments, exercise could serve as an alternative approach to SSRIs or could be integrated with SSRI-driven methods. Therefore, this study has aimed to investigate the effects of exercise, two escitalopram doses, and exercise-accompanied escitalopram on synaptic potency and long-term plasticity in the hippocampal CA1 area in rats with chronic stress.

## Materials and Methods

***Animals***

Seventy-two male Wistar rats (200–250 g) were purchased from the animal nest in the Faculty of Pharmacy, Isfahan University of Medical Sciences, Iran. The rats were under a 12-hour light/dark cycle (lights on at 07:00) at constant temperature (23±2 °C) and humidity (55±5%). These rats received food and water *ad* *libitum*. Also, before the experimental procedures, they were acclimatized to the standard conditions for a week. All experiments were performed in compliance with the Ethics Committee of Animal Use and were approved by it (IR.MUI.MED.REC.1398.607). The rats were restrained 6 hr/day for 14 days and different treatment protocols were applied during the next 14 days. At the beginning of the study, the animals were randomly assigned to nine equal groups (*n*=8):

1) Control (Co) group, in which the rats were transferred to the laboratory and received no special treatments.

2) Sham Exercise (Sh.Exe) group, in which the rats were placed on the turned-off treadmill apparatus (1 hr/day) for the next 14 days.

3) Sham Injection (Sh.Inj) group, in which the rats received equal volumes of normal saline (drug vehicle) for the next 14 days.

4) Chronic Restraint Stress (CRS) group, in which the rats were exposed to restraint stress of 6 hr/day for 14 days.

5) Chronic Restraint Stress-Exercise (CRS-Exe) group, in which the rats were first exposed to restraint stress of 6 hr/day for 14 days, and then received regular exercise 1 hr/day for the next 14 days.

6) Chronic Restraint Stress-Escitalopram10 (CRS-Esc10) group, in which the rats were exposed to restraint stress of 6 hr/day for 14 days, and then received escitalopram at a dose of 10 mg/kg/day for the next 14 days. 

7)  Chronic Restraint Stress-Escitalopram20 (CRS-Esc20) group, in which the rats were exposed to restraint stress of 6 hr/day for 14 days, and then received escitalopram at a dose of 20 mg/kg/day for the next 14 days. 

8) Chronic Restraint Stress-Escitalopram10-Exercise (CRS-

Esc10-Exe) group, in which the rats were exposed to restraint stress of 6 hr/day for 14 days, and then simultaneously received escitalopram at a dose of 10 mg/kg/day and regular exercise for the next 14 days.

9) Chronic Restraint Stress-Escitalopram 20-Exercise (CRS-

Esc20-Exe) group, in which the rats were exposed to restraint stress of 6 hr/day for 14 days, and then simultaneously received escitalopram at a dose of 20 mg/kg/day and regular exercise for the next 14 days. 

***Induction of chronic restraint stress***

Chronic restraint stress is a common animal model to induce emotional stress, anxiety, depression, and their associated physiological changes (19). In this study, chronic restraint stress (6 hr/day, 8:00−14:00) was induced for 14 days (20); therefore, each rat was separately placed in a transparent plastic cylinder.

***Drug treatment***

Escitalopram was administered by the intraperitoneal (IP) injections of pure escitalopram oxalate powder (10 and 20 mg/kg; Sobhan-Daru Co., Iran), dissolved in the sterile normal saline (0.9%) for 14 consecutive days (21).

***Exercise treatment***

The rats were subjected to a 1 hr/day treadmill (Maze router apparatus, Tabriz, Iran) running for 14 consecutive days in compliance with a previous study protocol (3). The exercise speed was 20–21 m/min at a zero-degree (0º) slope. The animals were familiarized with the treadmill running 3 days before the experiment. They were forced to run at the speed of the treadmill and received a mild electric shock (about 0.3 mA) from the grid at the back of the apparatus. Electric shocks were used sparingly to promote running. These mild shocks were applied at the beginning of the experience and then discontinued to avoid any pain stress after familiarization. During the remaining exercise period, the treadmill running was completed without shock to the animals.

***Electrophysiological procedures***

**Figure 1:**
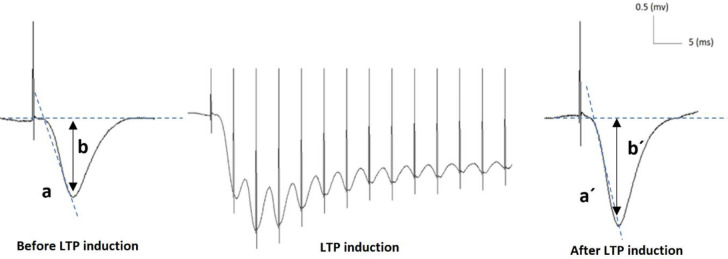
A schematic representation of the changes in the slope and amplitude of fEPSP before and after LTP induction by high-frequency stimulation in the control group in Wistar rats: (a) and (a´) fEPSP slope; (b) and (b´) fEPSP amplitude

LTP: Long-term potentiation; fEPSP: Field excitatory postsynaptic potential

The electrophysiological procedures complied with the protocols described in previously published studies (3, 22). The rats were anesthetized with urethane (1.5 g/kg, IP; Sigma-Aldrich Co., USA), and then placed in a stereotaxic frame (Stoelting Co., USA). A bipolar stimulation electrode (Teflon-coated stainless steel, diameter: 0.125 mm; Advent, UK) was placed in the right hippocampal Schaffer collateral pathway (AP=−4.2 mm; ML=3.8 mm; DV=−2.7 to −3.8 mm) and a unipolar recording electrode (Teflon-coated stainless steel) was moved to the right CA1 area from the upper left at an angle of 52.5° (AP=−3.4 mm; ML=1.5 mm; DV=−4.4 to −5.1 mm) until the maximal response was received. To minimize brain tissue trauma, the electrodes were inserted slowly (2 mm/min). Proper implantation of these electrodes in the correct position was confirmed by considering the physiological and stereotaxic indicators. Notably, the electrophysiological experiments were performed a day after the last day of the experiment (Day 29). Extracellular recording of the field excitatory postsynaptic potential (fEPSP) waveform is a commonly used technique for studying synaptic plasticity and long-term potentiation (LTP). Therefore, the fEPSP indices (i.e., slope and amplitude) were used as the preferred criteria for measuring synaptic plasticity (23). The fEPSP slope has been defined as the slope between the baseline and peak of the negative wave; however, the fEPSP amplitude is measured as the voltage difference between the negative peak and baseline of the fEPSP wave (3, 22) (Figure 1).

Henceforth, a stimulation of 0.1 Hz in the CA1 area evoked fEPSPs. As such, these potentials were amplified (×10^3^) and 1–3 kHz band-pass filtered. Subsequently, the signals were transferred through an analog-to-digital interface (Science Beam-D3111, eProbe Experiment software) to a computer. The obtained data were analyzed using the eTrace analysis software (Science Beam; Parto Danesh, Tehran, Iran). The stimulus-response or input-output (I/O) functions were acquired to verify if the interventions had influenced the basal circuity properties and synaptic potency (excitability) in the intended areas. As such, a systematic variation of the current stimulus (100–1000 μA) was used before LTP induction. After verifying the steady state of baseline fEPSP responses, recordings were obtained 30 min before and 60 min after LTP induction (Figure 1). Any alterations in the synaptic responses of the CA1 neurons could be determined this way. High-frequency stimulation (HFS) protocol of 100 Hz (4 bursts of 50 stimuli, 0.15 ms stimulus duration, at 10 sec inter-burst intervals) was used to induce LTP. The stimulation intensity was adjusted to approximately 50% of the maximum fEPSP slope response after recording the I/O functions. LTP was indicated as the initial baseline value percentage which was monitored after 60 min tetanic stimulation. It was measured by the varying initial fEPSP slope and amplitude values.

***Assessing serum corticosterone levels***

The rats were decapitated a day after the final session (Day 29, 16:00–18:00) of the electrophysiological recording procedures. After collecting the blood samples from the trunk, the serum samples were separated (centrifuged for 20 min, 6000 rpm) and stored at −80 °C for subsequent analyses. Also, the serum corticosterone levels were evaluated by a commercial enzyme-linked immunosorbent assay (ELISA) kit (Zellbio, Germany).

***Statistical analyses***

The obtained electrophysiological data were compared in all experimental groups via repeated-measures analysis of variance (ANOVA) and Tukey’s *post hoc* test. Serum corticosterone levels were analyzed by one-way ANOVA and Tukey’s *post hoc* test, and calculated in SPSS statistics software (v.24). In this study, the *P*-values less than 0.05 (*P*<0.05) were statistically significant. All data were expressed as means±SEM.

## Results

The control (Co) and both sham (Sh.Exe and Sh.Inj) groups presented no significant differences. Therefore, the Co group became the frame of reference for all comparisons in this study.

***Input-Output (I/O) functions***

**Figure 2:**
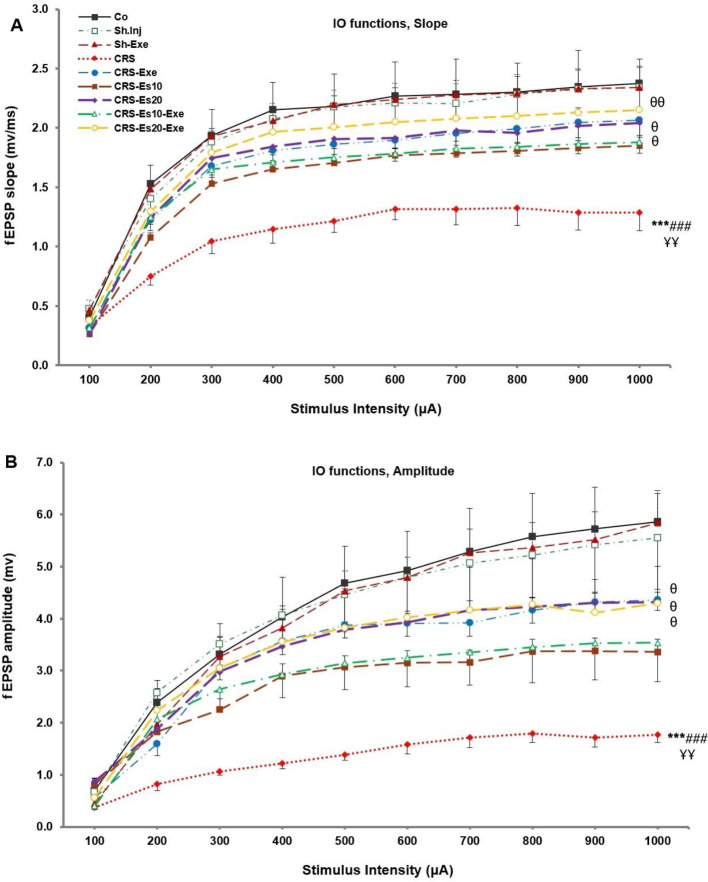
Input-output (I/O) curves of fEPSP (A) slope and (B) amplitude in the CA1 region for different experimental groups. The input-output (I/O) curves show the responsiveness of the apical dendritic layer and synaptic potency (excitability). Results are expressed as means±SEM (ANOVA test, Tukey’s *post hoc* test); ***P<0.001 compared with the Co group; ###*P*<0.001 compared with the Sh.Inj group; *P*<0.01 compared with the Sh.Exe group; *P*<0.05 and *P*<0.01 compared with the CRS group

**The Chronic Restraint Stress** (CRS) group showed significant (both* P*<0.001) decreases in the fEPSP slope and amplitude of their associated I/O curves compared with the Co group; this result indicated that CRS had reduced synaptic potency of the hippocampal CA1 apical dendritic layer. However, there was no significant difference in any of the other experimental groups in comparison with the Co group (Figure 2).

The fEPSP slope and amplitude of the I/O curves in the CRS-Esc10 and CRS-Esc10-Exe groups exhibited no significant difference compared with the CRS group (Figure 2). However, the fEPSP slope and amplitude presented significant enhancements in the CRS-Exe (both *P*<0.05), CRS-Esc20 (both *P*<0.05), and CRS-Esc20-Exe (*P*<0.01 and *P*<0.05, respectively) groups in comparison with the CRS group. Hence, exercise and escitalopram at a dose of 20 mg/kg/day were almost equally effective; nonetheless, the exercise-accompanied escitalopram20 had a partially non-significant therapeutic effect on the synaptic potency of the CA1 region (Figure 2).

***Long-term potentiation (LTP)***

**Figure 3:**
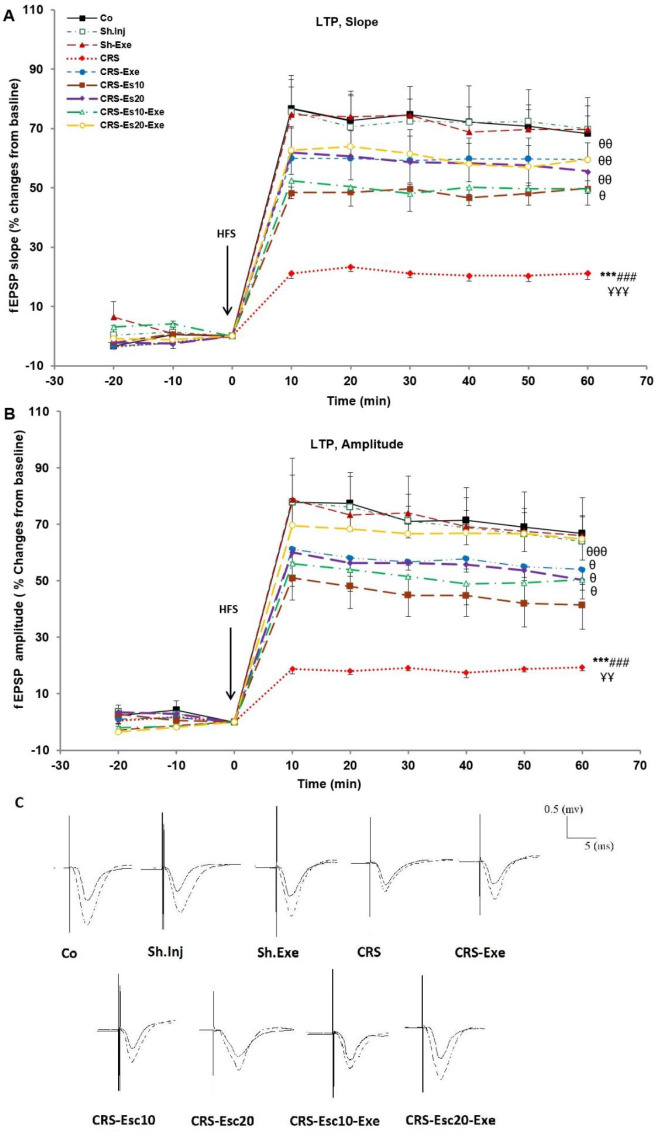
Effects of different treatment protocols on LTP induction in the CA1 region using 100 Hz high-frequency stimulation. (A) Changes in the fEPSP slope as percentages of the baseline response. (B) Changes in the fEPSP amplitude as percentages of the baseline response. (C) Sample traces of typically recorded fEPSPs in the hippocampal CA1 neurons before and after HFS induction for the LTP in all experimental groups. Results are expressed as means±SEM (ANOVA test, Tukey’s *post hoc* test); ***P<0.001 compared with the Co group; ###*P*<0.001 compared with the Sh.Inj group; *P*<0.01 and *P*<0.001 compared with the Sh.Exe group; *P*<0.05, *P*<0.01, and *P*<0.001 compared with the CRS group

**The fEPSP slope and amplitude reduced significantly (** *P*<0.001 both) in the CRS group compared with the Co group, indicating poor LTP induction and maintenance in this group. Also, the fEPSP slope and amplitude after the LTP induction were non-significantly lower in the CRS-Esc10 and CRS-Esc10-Exe groups compared with the Co group (Figure 3). According to these results, although the aforementioned therapeutic approaches to chronic stress were partially effective, chronic stress had severely disrupted the LTP data to an extent that it could not re-attain the normal levels again. The effects of administering escitalopram at a dose of 10 mg/kg alone failed to reverse the CRS-induced memory impairments. However, chronic stress for 14 days might induce a state of depression that administration of escitalopram at a dose of 10 mg/kg alone seemed incapable of improving its slope and amplitude after the LTP induction. 

After the LTP induction, slope and amplitude had significant enhancements in the CRS-Exe (*P*<0.01 both), CRS-Esc20 (*P*<0.01 and *P*<0.05, respectively), CRS-Esc10-Exe (*P*<0.05 both) and CRS-Esc20-Exe (*P*<0.01 and *P*<0.001, respectively) groups in comparison with the CRS group. These results suggested that all of these treatment methods were beneficial for improving memory deficits in the chronically stressed group. However, there was no significant difference in the fEPSP slope and amplitude among these therapeutic groups, including the CRS-Exe, CRS-Esc20, CRS-Esc10-Exe, and CRS-Esc20-Exe groups concerning the LTP data (Figure 3). These results indicated that exercise accompanied by both doses of escitalopram, especially a 20 mg/kg dose of escitalopram, had a partial additive impact on memory improvement after the induction of chronic stress.

***Changes in serum corticosterone levels***

**Figure 4:**
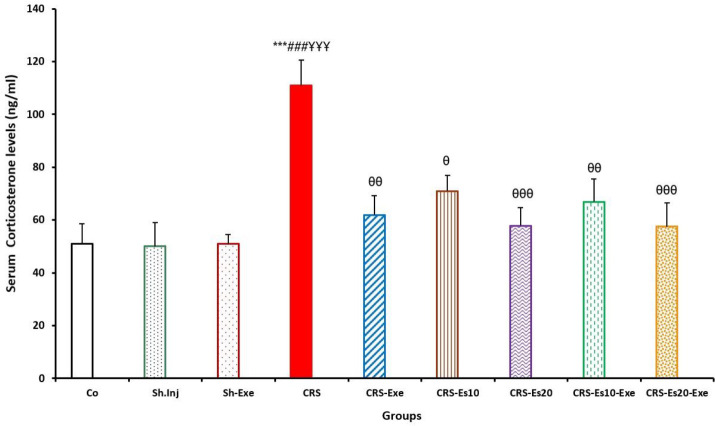
Changes in the serum corticosterone levels (ng/ml) in all experimental groups in Wistar rats. Results are expressed as means±SEM (one-way ANOVA followed by Tukey's *post hoc* test). ***P<0.001 compared with the Co group; ###*P*<0.001 compared with the Sh.Inj group; *P*<0.001 compared with the Sh.Exe group; *P*<0.05, *P*<0.01, and *P*<0.001 compared with the CRS group

As shown in Figure 4, serum corticosterone levels were significantly higher (*P*<0.001) in the CRS group compared with those in the Co group. These results indicate that CRS activated the HPA axis. In all treatment groups, serum corticosterone levels did not show any significant differences compared with the Co group. As seen in Figure 4, the serum corticosterone levels had significant differences in the CRS-Exe, CRS-Esc10, and CRS-Esc10-Exe groups compared with the CRS group (*P*<0.01, *P*<0.05, and *P*<0.05, respectively). In this figure, more significant decreases in the serum corticosterone levels were observed in the CRS-Esc20 and CRS-Esc20-Exe groups in comparison with the CRS group (*P*<0.001 both). These results indicated that exercise simultaneous with receiving either dose of escitalopram administration slightly contributed to reducing serum corticosterone levels.

***Brain tissue histology***

**Figure 5:**
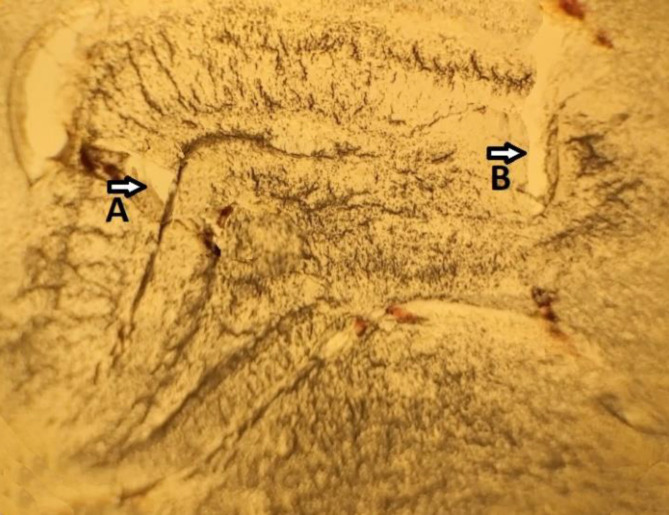
An electrode site sample in (A) the CA1 region and (B) the Schaffer collateral pathway of the hippocampus by observing tissue incision (2.5*10 magnified)

The primary clue about the stimulation and recording sites in the hippocampus is the field potential pattern. In this study, these sites were histologically verified (further assurance) by rapidly discarding and storing the brains of decapitated animals in 10% formalin for at least 3 days. For the histological confirmation, a freezing microtome was used for the frozen brain serial transverse sections (60 μm). Additionally, the injection sites were determined by a light microscope following the rat brain atlas (Figure 5).

Co: Control group, Sh.Inj: Sham injection group, Sh.Exe: Sham Exercise group, CRS: Chronic Restrain Stress group, CRS-Exe: Chronic Restrain Stress-Exercise group, CRS-Esc10: Chronic Restrain Stress-Escitalopram 10 mg/kg group, CRS-Esc20: Chronic Restrain Stress-Escitalopram 20 mg/kg group, CRS-Esc10-Exe: Chronic Restrain Stress-Escitalopram 10-Exercise, CRS-Esc20-Exe: Chronic Restrain Stress-Escitalopram 20-Exercise group

Co: Control group; Sh.Inj: Sham injection group; Sh.Exe: Sham exercise group; CRS: Chronic restrain stress group; CRS-Exe: Chronic restrain stress-exercise group; CRS-Esc10: Chronic restrain stress-escitalopram 10 mg/kg group; CRS-Esc20: Chronic restrain stress-escitalopram 20 mg/kg group; CRS-Esc10-Exe: Chronic restrain stress-escitalopram 10-exercise; CRS-Esc20-Exe: Chronic restrain stress-escitalopram 20-exercise group; LTP: Long-term potentiation; HFS: High-frequency stimulation; fEPSP: Field excitatory postsynaptic potential

Co: Control group; Sh.Inj: Sham injection group; Sh.Exe: Sham exercise group; CRS: Chronic restrain stress group; CRS-Exe: Chronic restrain stress-exercise group; CRS-Esc10: Chronic restrain stress-escitalopram 10 mg/kg group; CRS-Esc20: Chronic restrain stress-escitalopram 20 mg/kg group; CRS-Esc10-Exe: Chronic restrain stress-escitalopram 10-exercise; CRS-Esc20-Exe: Chronic restrain stress-escitalopram 20-exercise group

## Discussion

The effects of exercise, different doses of escitalopram (10 and 20 mg/kg), and exercise-accompanied escitalopram (both doses) were investigated on synaptic potency and LTP of the hippocampal CA1 area in rats that were exposed to chronic stress. 

Chronic stress reduced synaptic potency and the intensity of long-term plasticity in the hippocampal CA1 area. Also, chronic stress increased serum corticosterone levels. A decrease in synaptic potency was previously attributed to the reduced proliferation rate (of new cells) due to chronic stress (24). Moreover, chronic stress inhibits synaptic flexibility and LTP in the hippocampal-nucleus accumbens pathway (25). Nevertheless, different mechanisms could be associated with the impairment of synaptic potency and long-term plasticity in chronically stressed subjects. For instance, chronic stress adversely affects N-methyl-D-aspartate (NMDA) receptors, hyperpolarization amplitude, calcium influx, and glutamate synaptic transmission (26, 27). These factors may subsequently influence the membrane potential and LTP capability through hormonal and biochemical changes, as well as alterations of neurotransmitters (26, 28). In this regard, some studies have shown that increased corticosterone levels caused changes in cerebral plasticity, which might in turn lead to structural changes in the hippocampus (29, 30). According to some *in vivo* and *in vitro* studies, LTP was impaired in the hippocampal neurons after exposure to high doses of corticosteroids (31). 

Based on present findings, exercise therapy in chronically stressed subjects improved synaptic potency, LTP induction, and maintenance, as well as serum corticosterone levels. Both forced and voluntary exercise models increase LTP and synaptic strength (32, 33), probably by activating the serotonergic neurons and increasing their firing rate (34). It is reported that exercise would ameliorate various brain functions, such as increasing antioxidant defense, activating synaptic plasticity mechanisms, and enhancing serotonergic neuronal activity, neurogenesis, and metabolic capacity (34-37). Moreover, the exercise duration was effective in the LTP responses (38). However, forced exercise over a short period could be regarded as a stressor that adversely affects memory and even corticosterone levels (39, 40).

Based on the present data, escitalopram at a dose of 10 mg/kg alone did not have any significant therapeutic impact on the cellular memory mechanisms in chronically stressed subjects. Exposure to chronic stress for 14 consecutive days could possibly induce a state of depression such that administration of escitalopram at a dose of 10 mg/kg alone could not significantly improve LTP as a cellular model of memory. However, a 20 mg/kg dose of escitalopram alone improved synaptic potency and long-term plasticity in the stressed subjects. A study reported that a 20 mg/kg dose of escitalopram ameliorated protein markers (like BDNF and synaptophysin) involved in hippocampal synaptic plasticity (41, 42). Popoli *et al*. (2008) demonstrated that administration of escitalopram at a dose of 25 mg/kg reduced LTP under stress (43). Additionally, both doses of escitalopram (particularly the 20 mg/kg dose) decreased serum corticosterone levels more significantly. Therefore, administration of escitalopram might have positively affected the regeneration process for the damaged dendrites in the prefrontal cortex and hippocampus (41, 44). It is reported that administration of 10 mg/kg escitalopram in the chronic stress model reversed LTP impairment and reduced corticosterone levels in the stressed subjects (45, 46). Another study has similarly indicated the potential impairment of LTP induction due to the escitalopram dose of 10 mg/kg (10). Escitalopram could not improve the recognition memory in the serotonin-depleted rats (47); also, it could not enhance the hippocampal BDNF levels in the stressed rats (48). As such, these doses of escitalopram did not affect NMDA receptors in rodents under chronic stress (49). Hence, various signaling pathways seem to be associated with the efficacy of different escitalopram doses in the brain (50). According to some studies, escitalopram is a dose-dependent drug because the increase in the drug dosage has enhanced serotonin concentrations (51). Moreover, the serotonergic system positively affected LTP induction and maintenance (52). It is proposed that increasing the drug dosage might positively contribute to the treatment of memory disorders (53). Overall, the outcome of drug usage may depend on the type, time, and duration of drug treatment as well as the presence of stress, type of stress induction, and drug usage (54). Furthermore, the difference between these two escitalopram doses might be related to an initiation delay in the manifestation of SSRI-related therapeutic effects. For instance, desensitization or neuroadaptation of monoamine receptors, and especially serotoninergic auto-receptors in the brain, may lead to such types of delayed reactions (55). Moreover, increased serotonin levels due to a sustained administration of antidepressants led to down-regulation or desensitization of these receptors (55). Nevertheless, higher doses of escitalopram seem more potent in increasing and maintaining serotonin (51). While escitalopram at a dose of 10 mg/kg had a 2-week interval for neural adaptation in the rat’s brain, neuroadaptation occurred faster with its higher dose (20 mg/kg) (56). 

Based on other data, the combined effects of escitalopram (10 and 20 mg/kg) and exercise improved long-term plasticity and serum corticosterone levels in subjects with chronic stress. In this study, its positive effects were not significant compared with the singular treatment methods (by escitalopram 10 and 20 mg/kg or exercise). In the present study, exercise accompanied both doses of escitalopram (10 mg/kg and particularly 20 mg/kg) and had a partial additive impact on improving synaptic plasticity in the chronically stressed subjects. Therefore both treatment models not only could effectively increase BDNFs (35, 42) and serotonin (34, 51) but also possibly decrease corticosterone levels in the brain (38, 41). Serotonin plays a key role in memory and regulates important memory-related neurotransmitters, such as GABA and glutamate (57). An SSRI-related increase in serotonin levels leads to activation of the HPA axis due to different serotonin receptors in the hypothalamus (58). Moreover, desensitization of serotonin receptors results in an activity-driven decrease of these receptors over the injection period (58). Hence, with a 10 mg/kg escitalopram dose longer duration was required for desensitization of serotonin receptors and increasing the serotonin levels in the brain (55, 56). Therefore, exercise seems to contribute to both doses of escitalopram in serotonin changes. In addition, memory improvement could develop further if the exercise period increases (38). The onset of treatment response within a 2-week interval could be an important indicator for the subsequent responses (59). In this study, escitalopram administration at both doses of 10 and 20 mg/kg produced proper therapeutic effects; moreover, when accompanied by exercise, these escitalopram dosages had a partial additive impact on memory improvement over the 2-week study period. Based on a previous study, the beneficial effects of exercise were time-dependent in stress conditions (60). However, present findings indicate that treatment continuation using both escitalopram dosages and exercise could provide a desirable additive response for memory improvement in chronically stressed subjects.

## Conclusion

In the hippocampal CA1 areas, synaptic potency and long-term plasticity were severely impaired by chronic stress. Also, serum corticosterone levels were increased by chronic stress. Exercise, escitalopram (20 mg/kg), and exercise-accompanied escitalopram (10 and 20 mg/kg) improved synaptic potency and long-term plasticity and had a partial additive impact on memory improvement in chronically stressed subjects. However, escitalopram 10 (only with exercise) was able to reverse memory deficits in chronically stressed subjects. Therefore, to develop combined methods using both escitalopram and exercise, further molecular and biochemical research regarding neuroplasticity is required. The potential implications of such methods in the treatment of depression-induced memory deficits should be thoroughly understood.

## Authors’ Contributions

MR Designed the experiments and MZ Performed them. MR Analyzed the data. All authors contributed to writing the manuscript and approved its final version.

## Funding

This work was supported by the Isfahan University of Medical Sciences, Iran.

## Ethical Statment

All experiments were approved by the Research and Ethics Committee of Isfahan University of Medical Sciences (IR.MUI.MED.REC.1398.607).

## Conflicts of Interest

The authors declare no conflicts of interest.
